# Early evidence for emotional play contagion in juvenile ravens

**DOI:** 10.1007/s10071-020-01466-0

**Published:** 2021-01-09

**Authors:** Katharina Wenig, Palmyre H. Boucherie, Thomas Bugnyar

**Affiliations:** 1 Department of Behavioral and Cognitive Biology, University of Vienna, Althanstrasse 14, 1090 Vienna, Austria; 2 Haidlhof Research Station, University of Vienna and University of Veterinary Medicine Vienna, 2540 Bad Vöslau, Austria

**Keywords:** Emotional contagion, Behavioral contagion, Play behavior, Cognition, Ontogeny, Empathy

## Abstract

Perceiving, evaluating and reacting towards conspecifics’ emotional states are important challenges of social group living. Emotional contagion describes an alignment of emotional states between individuals and is widely believed to be based on behavioral synchronization, i.e., behavioral contagion. As basic empathy-like processes, the occurrence of both forms of contagion seems to underlie early ontogenetic trajectories in humans and non-human species. In the present study, we assessed play as a context for studying the development of emotional contagion and its interlink with behavioral contagion in ten juvenile common ravens. Ravens are exceptional players that engage in all three forms of play: object, locomotion and social play. To assess potential ontogenetic patterns of both behavioral and emotional contagion, we tested juvenile ravens at two different periods of early development, at three- and six-month post-hatching. We elicited object play in one or several ravens (demonstrators) in a standardized experimental environment, using a playground setup. At both test ages, we found evidence for emotional contagion as observer ravens showed an increase of locomotion and social play after we provided the demonstrator(s) with the playground setup, but no significant changes in the amount of object play. Hence, observers did not copy motor patterns from demonstrator(s) but engaged in other forms of play. Our findings speak for a transfer of a general mood state in the context of play in ravens as young as 3 months and against behavioral mimicry as a precondition for emotional contagion.

**Supplementary Information:**

The online version contains supplementary material available at 10.1007/s10071-020-01466-0.

## Introduction

Empathy is the ability to be affected by, share and understand other’s emotional states and perspectives (de Waal 2008). It is believed to be vital for the formation and maintenance of functioning human bonds and societies as it promotes smooth communication, coordination as well as group cohesion and prosocial behaviors (de Waal 2008, 2013; Decety 2015; Decety et al. 2016; Lamm, Rütgen, Wagner 2019). Like in human societies, well-functioning social interactions and resulting social bonds can also lead to positive outcomes in non-human animal societies (Kummer 1978; Cheney, Seyfarth, Smuts, 1986; fitness: Silk, Alberts, Altmann 2003; infant survival: Silk 2007; reproductive success: Cameron, Setsaas, Linklater 2009; adult longevity: Barocas et al. 2011). Therefore, it has been argued that the capacity for empathic-like behaviors should be beneficial for both humans and non-human animal species.

According to Preston and de Waal’s perception action model (2002), empathic-like behaviors range from basic processes, such as mimicry and contagion (emotional empathy) to more complex processes, such as perspective taking and targeted helping (cognitive empathy). While higher forms of empathic-like behaviors have been suggested for great apes (e.g., *Pan troglodytes, Pan paniscus)* on the basis of observational and experimental studies (consolation/reconciliation: de Waal and van Roosmalen 1979; Clay and de Waal 2013; Koski 2015; perspective taking: Krupenye et al. 2016; targeted helping: Yamamoto, Humle, Tanaka 2012), the underlying motivation of these behaviors and their phylogenetic distribution are anything but clear (see debates about targeted helping in rats, (*Rattus norvegicus*: Sivaselvachandran et al. 2018); and dogs (*Lupus familiaris*: Bräuer, Schönefeld, Call 2013). Hence, besides expanding studies on cognitive empathy towards other animal species, a systematic and thorough assessment of the emotional basis would add to an understanding of a species’ general capability for empathy-like behaviors.

In Preston and de Waal’s model, a widely accepted theory (Preston and de Waal 2002), one building block of empathy is emotional contagion, while the basis of emotional contagion is behavioral contagion—an unconscious and automatic mimicry and matching of movements, gestures and facial expressions (Hatfield, Cacioppo. Rapson 1994; Chartrand and van Baaren 2009). Behavioral contagion has been widely studied in both humans (Chartrand and van Baaren 2009) and non-human species. For instance, primates have been observed to show contagious scratching (*Macaca mulatta*: Feneran et al. 2013; *Macaca fuscata*: Nakayama 2004) and contagious scent marking (*Callithrix jacchus*: Massen, Šlipogor, Gallup 2016), budgerigars engage in contagious stretching (*Melopsittacus undulates*: Miller et al. 2012) and mice show contagious itch (*Mus musculus*: Yu et al. 2017). Furthermore, primates (*Pan troglodytes*: Anderson, Myowa-Yamakoshi, Matsuzawa 2004; Campbell et al. 2009*;* Theropithecus gelada: Palagi et al. 2009), budgerigars (*Melopsittacus undulates*: Miller et al. 2012) and dogs (*Lupus familiaris*: Joly-Mascheroni, Senju, Shepherd 2008 but see Harr, Gilbert, Phillips 2009 and O’Hara and Reeve 2011) yawn contagiously, some of them even across species. The phenomenon of contagious yawning was originally studied in humans, both clinically (e.g., individuals with autism spectrum disorders: Senju et al. 2007) and ontogenetically (Anderson and Meno 2003; Helt et al. 2010). In line with the results in humans that contagious yawning could not be reliably elicited before preschool age, yawning was contagious for adult, but not for infant individuals in two primate studies (*Pan troglodytes*: Anderson et al. 2004; *Theropithecus gelada:* Palagi et al. 2009). Hence, basic empathy-like processes like yawn contagion seem to rely on ontogenetic developments both in humans and non-human species.

Building on behavioral contagion, the concept of emotional contagion refers not only to an alignment of postures and movements (Hatfield et al. 1994) but also to an alignment of emotional states. Unlike the vast literature on behavioral contagion, potential transfers of emotions as a result of behavioral synchronization have been studied less in animal species (for a review see Adriaense et al. 2020), probably due to difficulties in the assessment of animals’ emotional states in general, and emotional contagion in particular. Experimental studies that did address emotional contagion in animals typically used stimuli of negative valence to elicit affective reactions in a demonstrator individual, to see if those affective states would consequently transfer to an observer individual. Research in rodents for example, showed an increase in pain sensitivity (*Mus musculus:* Langford et al. 2006), distress-like behaviors (*Peromyscus maniculatus:* Kavaliers, Choleris, Colwell 2001), emotional arousal (*Rattus norvegicus domestica*: Knapska et al. 2010; Meyza and Knapska 2018), and increased anxiety (*Rattus norvegicus domestica:* Burman et al. 2007) in observer mice after witnessing a conspecific in pain or distress, or after hearing corresponding vocalizations. Further research elaborated on the elevating effect of kinship, familiarity and dominance on the expression of emotional contagion (Kavaliers, Colwell, Choleris 2005; Langford et al. 2006; Jeon et al. 2010; Jeon and Shin 2011; Martin et al. 2015). It has been argued that negative emotions are easier and more automatically transferred between individuals than positive information (Kelly, Iannone, McCarty 2016) as the former convey more important, potentially life-threatening information. However, recent behavioral experiments on domestic pigs (*Sus scrofa*) found support for contagion effects of both negative and positive affective states, such as ‘distress’ and ‘pleasure’ (Reimert et al. 2017).

Probably the best indications for transfers of positive emotional states come from studies on non-human animal play. Studies on captive rats revealed that social play increases when a playful individual is introduced to a less playful one (Pellis and McKenna 1992; Varlinskaya, Spear, Spear 1999) and studies on free-ranging kea (*Nestor notabilis)* showed an increase in playful behaviors after hearing playbacks of play vocalization (Schwing et al. 2017). Furthermore, primates, like orangutans (*Pongo pygmaeus:* Davila Ross, Menzler, Zimmermann 2007), gelada baboons (*Theropithecus gelada:* Mancini, Ferrari, Palagi 2013) and tonkean macaques (*Macaca tonkeana:* Scopa and Palagi 2016) have been observed to rapidly mimic their play partners’ facial expressions and/or to engage in longer play bouts when rapid facial mimicry was expressed. Hence, it has been argued that the mimicry of play faces can evoke similar positive affective states in both demonstrator and observer (Mancini et al. 2013). However, most of the previously mentioned studies on animal play, strictly speaking, showed behavioral contagion (e.g., mimicry of facial expressions) and concluded an effect on the observer’s emotional state without duly testing for it.

To separate emotional contagion from behavioral contagion, Osvath and Sima (2014) introduced the idea of using the variety of motor patterns shown during play (e.g., Burghardt 2005). Specifically, they argued that “if a category of play in one individual induces a different category in another, this suggests the spread of a playful mood rather than released, species-specific behavior” (p. 198). In their study on sub-adult ravens (*Corvus corax*), subjects indeed engaged in various types of play behaviors as a response to a group member’s playful object manipulation. Osvath, Osvath and Bååth (2014) also described first signs of play contagion in three raven nestlings, but the exploratory character of that study does not allow to disentangle whether it was behavioral or also emotional contagion. In the present study, we followed up on the studies by Osvath and colleagues (2014) and experimentally tested i) for behavioral or emotional contagion during raven play and ii) whether contagion effects were dependent on age. We thus aimed to i) replicate Osvath and Sima’s findings on emotional contagion (2014) in younger ravens and ii) assess ontogenetic patterns of both forms of contagion, by testing juvenile ravens at two different periods of early development (three- and six-month post-hatching). To our knowledge, hardly any studies have experimentally and systematically addressed the developmental trajectories of behavioral and emotional contagion in non-human animals (but see observational studies in primates Anderson et al. 2004; Palagi et al. 2009). This is surprising as ontogenetic patterns may help us to understand how the two types of contagion effects are interlinked: if behavioral contagion occurs at earlier stages of development as emotional contagion, this would support the common assumption that emotional contagion builds on behavioral contagion (e.g., Preston and de Waal 2002). However, recent experiments indicate that emotional contagion can be expressed independently from behavioral contagion (Adriaense et al. 2019). Whether this is also true for young animals is unknown.

Ravens are an excellent choice for addressing questions about emotional contagion. They are highly responsive to the behavioral and emotional expressions of conspecifics (Adriaense et al. 2019), even when physically separated for the purpose of experimental testing. They are also renowned as exceptional players in terms of complexity, volume and innovativeness (Gwinner 1966; Ficken 1977; Bekoff and Byers 1998; Heinrich and Smolker 1998) and engage in all three categories of play: object play, locomotion play and social play (Gwinner 1966; Ficken 1977; Burghardt 2005). We here provided one (condition 1) or several (condition 2) demonstrator individuals with a playground setup to stimulate object play, while the remaining individual(s) were able to observe the demonstrator(s) through a wire mesh. If observers were affected by the demonstrator(s)’s actions on a behavioral level, we expected them to express similar motor patterns, i.e., show behavioral mimicry through an increase of object play. However, if observing others play led to an alignment on an emotional level, observers should get into a general mood to play and consequently engage in various categories of play, including locomotion or social play. While the logic of our study design followed that of Osvath and Sima (2014), we applied a more systematic experimental procedure by physically separating birds and eliciting object play in one or several demonstrators (conditions 1 and 2). The latter allowed us to evaluate whether the number of demonstrators affects the likelihood and strength of potential contagion effects.

Importantly, we focused on juvenile ravens in their first year and tested the subjects at two different ages during their early ontogeny (before and after their first summer, i.e., session 1—at three-month post-hatching and session 2—at six-month post-hatching) to pinpoint possible developmental effects. Previous ontogenetic studies on ravens’ socio-cognitive skills indicated a possible step in cognitive development at the end of the birds’ first summer: behaviors indicative of perspective taking, such as gaze following behind visual barriers (Schloegl, Kotrschal, Bugnyar 2007) and using barriers to hide from others’ view at caching (Bugnyar, Stoewe, Heinrich 2007), were hardly seen at three-month post-hatching (before summer) but present at six-month post-hatching (after summer). According to the observation that behavioral contagion is already present in raven nestlings (Osvath et al. 2014) and following the widely recognized definition of behavioral mimicry as a precursor of emotional contagion (Preston and de Waal 2002; Chartrand and van Baaren 2009; Feneran et al. 2013), we expected to find behavioral contagion in both test sessions, before and after summer. Following the assumption that emotional contagion represents a building block of empathy and builds on behavioral contagion, we expected it to develop later, and thus be present only after the subjects’ first summer, i.e., in the second test session.

## Methods

### Animals and housing

**Fig. 1:**
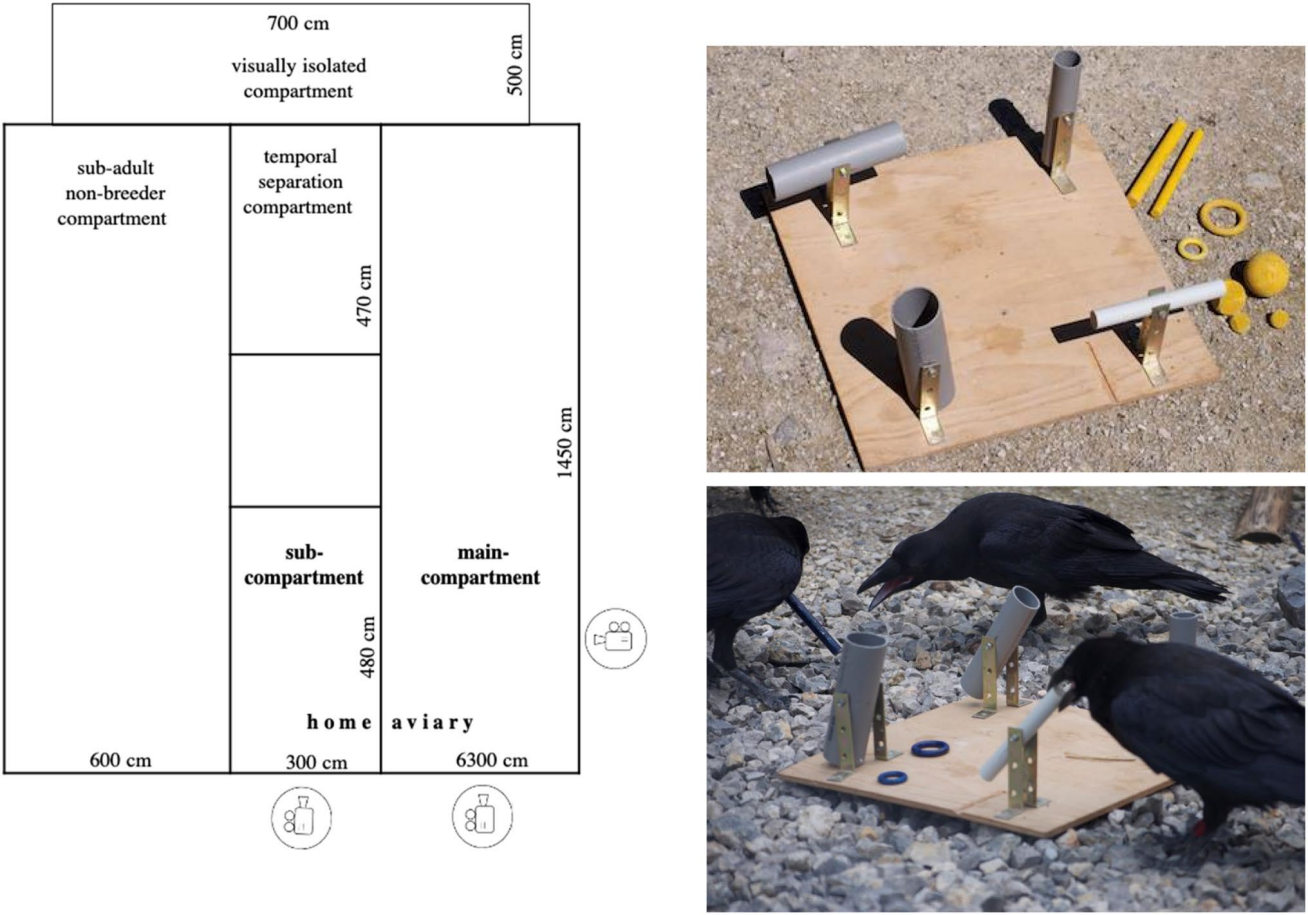
Left: sketch of keeping aviary, depicting compartment sizes, the sub- and main compartments and camera positions. Right top: playground setup (Auersperg et al. 2015; picture does not show complete set of freely movable objects). Right bottom: Three ravens interacting with the playground setup Photographs by S. Houszka

We tested a total of ten juvenile common ravens (*Corvus corax*; seven females, three males). All ravens hatched within a 1.5-week period in early April 2018 in our captive colony, at the Haidlhof research station, Bad Vöslau, Austria. After three weeks, they were removed from their family unit to be hand-raised by humans. After fledging (mid-May), the juvenile ravens moved to an outdoor aviary (928 m^2^). They were kept in one social group during their first year of life; afterwards, they were merged with sub-adults and adult non-breeders. This housing schedule should simulate natural social groupings of non-breeding ravens (Marzluff, Heinrich, Marzluff 1996; Braun and Bugnyar 2012). The aviary complex was also designed to allow temporal separation for experimental testing: it could be divided into four rooms by sliding doors (Fig. 1); in addition, the aviary was connected to a visually isolated compartment (35 m^2^). The juveniles’ aviary was adjacent to five other aviaries (not directly connected, but birds could hear and partially see each other depending on the aviary; each adjacent aviary 8 m × 10 m × 5 m; not depicted in Fig. 1) where older raven pairs were kept. All aviaries were equipped with natural structures (e.g., wood, rocks, gravel, sand) and artificial objects (e.g., food bowls, bathing pools, toys) to promote a variety of behavior expressions (e.g., exploration, manipulation, and caching of food and objects, conflict escape possibilities) and to provide protection during extreme weather conditions. Individuals were marked with a colored leg band for identification. They were kept on a food diet composed of meat, eggs, vegetables, dairy products, bread and phytobiotics. Water was available ad libitum.

### Experimental procedure

The first session of the experiment was carried out in late spring 2018, approximately three-month post-hatching and two weeks after the juvenile ravens moved to the outdoor aviary; the second session was carried out in autumn 2018, approximately six-month post-hatching. Both sessions took place before the juvenile ravens were merged with sub-adults and adult non-breeders. Prior to the experiments, all ravens got habituated to being physically separated in a sub-compartment (14.4 m^2^) of their home aviary, by closing a wire-mesh door. An experimental trial started with calling a single individual into that sub-compartment and closing the wire-mesh door, while the other group members remained in the adjacent main compartment (91.35 m^2^, Fig. 1). Note that the separated individual had full visual and acoustic access to its group members, and vice versa, throughout the experimental trial. The separated bird was given a two-minute cool-down phase before the actual trial started. An experimental trial consisted of a 10-min baseline phase and a 20-min experimental phase. In the baseline phase, the naturally occurring play behavior of all ten ravens (one separated individual and nine ravens in the social group) was video recorded without providing any additional play incentives. In the experimental phase, a playground setup (Auersperg et al. 2015) was introduced and, depending on the condition, placed either in the separation compartment to the separated individual (condition 1, making the group individuals the observers and the separated individual the demonstrator) or in the group compartment to the nine group individuals (condition 2, making the separated individual the observer and the group individuals the demonstrators). The longer duration of the experimental phase in comparison to the baseline phase was chosen to grasp the observer(s)’s full behavioral response once play behavior was triggered by providing a play incentive to the demonstrator(s).The playground setup consisted of a wooden board (40 × 40 cm) with four movable tubes attached and ten freely movable, colorful objects of different shapes (cubes, sticks, rings) that were placed around the board (Fig. 1). Individual play behavior of all ten ravens was again video recorded during the experimental phase. Three different camera angles were used to get a detailed view of the subjects’ behavioral patterns in both the sub- and main compartment: one camera recorded the sub-compartment while two cameras covered the bigger main compartment. Each individual was tested once per condition at both ages (i.e., at three-month and six-month post-hatching). Two video recordings (Kai—condition: 1, age: three-months; Janis—condition 2, age: three-months) were lost due to camera failure, resulting in a total number of 19 trials for both condition 1 and condition 2. To test for the effect of playground setup presentation itself, we randomly added five ghost control trials in each session (three- and six-month post-hatching). Here, the playground setup was also presented in the separation compartment during the experimental phase but without previously separating a demonstrator individual who could interact with the setup. This control condition aimed at ruling out the possibility that the respective play behaviors observed in the experimental phases in condition 1 and 2 would be a response to the playground setup presentation and not result from the observation of a conspecific engaging in object play with the playground setup.

### Ethical statement

All birds were captive-bred and taken into human care at an early age (between 18 and 25 days, i.e., when they had hardly any feathers and slept most of the day). They were kept in artificial nests in groups of two birds until fledging (i.e., six-seven weeks post-hatching) and were housed in one social group since. All individuals participated in experiments voluntarily and were never deprived of food or water. The described housing and testing conditions comply with the ASAB/ABS Guidelines for the Use of Animals in Research, the Austrian government guidelines, and the institutional guidelines of the University of Vienna. The study was approved by the Animal Ethics and Experimentation Board of the Faculty of Life Science, University of Vienna (2020-003). As this study was strictly non-invasive and based purely on behavioral tests, it is not classified as an animal experiment under the Austrian Animal Experiments Act.

### Video analysis

The resulting video material was coded using Solomon Coder (© András Péter) with regard to all three categories of play behavior (object play, locomotion play, social play (Gwinner 1966; Fickens^1977^; Heinrich and Smolker 1998; Osvath and Sima 2014). Object play was defined by the manipulation of natural or artificial objects that were available in the aviary (e.g., sticks, old shoes, toys); within the experimental phase, object play also encompassed the manipulation of the playground setup. We excluded cases when a bird started to peck on the same object intensively and repeatedly in a stereotypic way as this behavior has also been described as a reaction to an aversive situation (e.g., Munteanu et al. 2017). Locomotion play included play jumps (i.e., spontaneous jumps up or forward), play flights (i.e., repeated locomotion between the same locations in the aviary, excluding agonistic chasing), lying on the ground (i.e., on the body side, feet might move and/or grasp object), and hanging from elevated structures (such as perches or the ceiling net, held by beak or feet). Social play was defined as a playful interaction with one or more conspecifics, namely co-lying (i.e., lying next to each other), co-flying (i.e., repeated locomotion between the same locations in the aviary, aligned next to one another), and co-manipulation (i.e., manipulation of the same object, at the same time or in turns).

Three different camera angles were used to get a detailed view of the subjects’ behavioral patterns in both the sub- and main compartment: one camera recorded the sub-compartment while two cameras covered the bigger main compartment. The two resulting videos of the main compartment were coded successively and integrated into one overall coding sheet. All videos were coded by the main experimenter (KW). 10% of the video material was additionally revised by a second person who was familiar with the subject group but uninformed about the study and its objectives. The agreement of detection and subsequent classification of behaviours into the three different play categories (object-, locomotion-, social play) were substantial to excellent (Stempler and Tsai 2008) with high agreement ranging from 88 to 100%.

### Statistical data analysis

We tested whether observer ravens engaged in more object, locomotion and social play during the experimental phase when the demonstrator(s) had access to the playground setup compared to the baseline phase when no play initiative was provided. We investigated potential contagion effects in two directions: either from a single demonstrator to the observing group individuals (condition 1) or from several demonstrators within a group to an observing separated individual (condition 2) to investigate how the number of demonstrators would affect the likelihood of contagion effects to occur. Moreover, we included age as a factor to assess potential developmental trajectories of both behavioral and emotional contagion.

To estimate the effect of contagion, we fitted three Generalized Linear Mixed Models (GLMM; (Baayen 2008), one for each of the three play categories considered, namely object play (model 1), locomotion play (model 2), and social play (model 3), each used as the binary response variable (i.e., did not/did the respective behavior within respective phase). We added as fixed factors: the phase (with levels 'baseline phase’, when no individual had access to the playground setup, and 'experimental phase', when one or several demonstrator(s) had access to the playground setup); age (with levels ‘session 1: three-months’ and ‘session 2: six-months’); trial number (consecutive integers counting from one within each session with session 1 referring to the age of three-month post-hatching and session 2 referring to the age of six-month post-hatching), and the interaction between ‘age’ and ‘trial number’ to control for possible habituation as further fixed effects into the model. In the models considering locomotion and object play, we further included fixed effects for the condition (with levels 'condition 1′, multiple observers in a group setting, being confronted with a single demonstrator and 'condition 2′, single observer, being confronted with multiple demonstrators) and the interaction between ‘condition’ and ‘phase’ to account for the possibility that the extent of the contagious effect would vary between birds confronted with a single demonstrator or multiple demonstrators. Note that for the social play model (model 3), we only considered observers in the group setting (condition 1), since per definition social play requires more than one individual.

Each model was fitted using all behavioral data from observer(s) in the experimental phase, and the subsequent observer(s) in the baseline (i.e., group individuals in condition 1 or separated individual in condition 2). Hence, the data used to fit the model did not comprise any observations of demonstrators, i.e., birds that had access to the playground setup.

To avoid pseudo-replication and account for sources of non-independence in the data, we included a random intercepts effect for the identity of the observed individual and the ID of the trial (accounting for the fact that all birds being in the group had been observed). To keep type I error rate at the nominal level of 0.05, we included random slopes (Schielzeth, Forstmeier 2009; Barr et al. 2013) of all fixed effects present in the model, including the interactions, within bird ID (i.e., in the social play model random slopes of the factor ‘condition’ and its interaction with factor ‘phase’, were not included). For the object play and locomotion play model, we manually dummy-coded and then centered the factors ‘phase’ and ‘condition’ before entering them into the random slopes part of the model. We also included the correlations among random intercepts and slopes.

Initially we used the proportion time individuals spent with the respective play type as the response and tried to fit the models with a beta error distribution and logit link function (Bolker 2008). However, none of these models converged, probably since all three response variables were heavily dominated by zeroes (percent zeroes, locomotion: 83; object play: 92; social play: 94). We, therefore, decided to dichotomize the response (i.e., did not/did the respective behavior) and fit models with a binomial error distribution and logit link function (McCullagh ands Nelder 1989). To account for varying durations of baseline phase and experimental phase, we included phase duration (log-transformed) as an offset term (McCullagh and Nelder 1989) into these models.

To avoid cryptic multiple testing (Forstmeier and Schielzeth 2011), we compared each full model as described above with a respective null model lacking access and its interaction with group (locomotion and object play model). This test utilized a likelihood ratio test (Dobson 2002). To test individual fixed effects, we dropped them from the full model, one at a time, and compared the resulting reduced model with the respective full model using a likelihood ratio test (Barr et al. 2013).

Prior to fitting the models, we z-transformed trial number to a mean of zero and a standard deviation of one, and centered session to a mean of zero to ease model convergence and enhance the interpretability of the model coefficients (Schielzeth 2010). We fitted the models in R (version 3.6.1; R Core Team 2019) using the function glmer of the package lme4 (version 1.1-21; Bates, Maechler, Bolker, Walker 2015) for models with binomial error distribution and the function glmmTMB of the equally named package (version 0.2.3; Brooks et al. 2017) for models with a beta error distribution. The sample sizes for these models were 738 total observations of ten individuals in 92 trials (locomotion model and object play model), and 666 total observations of ten individuals in 74 trials (social play model).

## Results

### Descriptive analysis of play behavior

Table 1 summarizes individuals’ play behavior. Note that each experimental trial consisted of a baseline phase (10 min), during which the behavior of all ravens was recorded in the absence of the playground setup; and an experimental phase (20 min), during which one or more individuals (physically separated) had access to the playground setup and the observers’ behavior was recorded. The test conditions were defined by who had access to the playground setup: Condition 1, featuring a single demonstrator and multiple observers; condition 2, featuring a single observer and multiple demonstrators. We tested at two different times during the subjects’ early ontogeny, at three-month (session 1) and six-month (session 2) post-hatching. The overall data set is composed of a total number of 38 trials.

**Table 1:** Average play behaviors in seconds (and corresponding standard deviations, minimum and maximum values and proportions per minute of the respective phase; format: Mean (SD/Min/Max/**Proportion**)) of all observers or demonstrators across all test trials for the respective conditions and sessions

Observer behavior	Condition 1	Condition 2	
Object play			
Baseline phase	3.10 (11.8/0/70.4/**0.31**)	6.78 (17.2/0/49/**0.68**)	Session 1
	6.51 (30.2/0/228/**0.65**)	4.88 (14.1/0/45/**0.49**)	Session 2
Experimental phase	7.47 (31.5/0/252.2/**0.37**)	1.68 (4,76/0/13.4/**0.08**)	Session 1
	3.90 (13.7/0/86/**0.2**)	7.3 (13.4/0/40/**0.37**)	Session 2
Locomotion play			
Baseline phase	0.8 (2.5/0/13.6/**0.08**)	0.88 (2.2/0/6.4/**0.09**)	Session 1
	0.79 (2.1/0/13.4/**0.08**)	3.18 (6.6/0/19/**0.32**)	Session 2
Experimental phase	3.01 (5/0/25.6/**0.15**)	2.03 (5.1/0/14.6/**0.1**)	Session 1
	2.85 (8.23/0/61.8/**0.14**)	3.12 (4.7/0/13/**0.16**)	Session 2
Social play			
Baseline phase	0.08 (0.37/0/2.2/**0.01**)	NA**	Session 1
	0.08 (0.8/0/8/**0.01**)	NA**	Session 2
Experimental phase	2.82 (9.5/0/56.8/**0.14**)	NA**	Session 1
	2.21 (7/0/35/**0.11**)	NA**	Session 2

Demonstrator behavior	Condition 1	Condition 2	
PG* Manipulation			
Experimental phase	193.63 (161.2/42.2/435.2/**9.68**)	104.33 (152.8/0/647/**5.22**)	Session 1
	269.6 (173.7/9/499/**13.48**)	150.85 (175.5/0/834.6/**7.54**)	Session 2
Object play			
Experimental phase	7.89 (23.7/0/71/**0.39**)	4.43 (12.4/0/64.6/**0.22**)	Session 1
	0.36 (1.1/0/3.6/**0.02**)	1.52 (9.2/0/87/**0.08**)	Session 2
Locomotion play			
Experimental phase	1.98 (4/0/12.4/**0.1**)	2.25 (4.7/0/26/**0.11**)	Session 1
	0.96 (0.9/0/2/**0.05**)	2.42 (6.8/0/58/**0.12**)	Session 2
Social play			
Experimental phase	NA**	0.95 (2.8/0/12.4/**0.05**)	Session 1
	NA**	0.28 (1.4/0/8/**0.01**)	Session 2

Baseline phase lasted 10 min, experimental phase lasted 20 min. Condition 1: playground setup (PG*) placed in separation compartment during the experimental phase, demonstrator = single individual, observer = group individuals. Condition 2: playground setup placed in group compartment during the experimental phase, demonstrator = group individuals, observer = single individual. Session 1 was carried out at three-month post-hatching and session 2 at six-month post-hatching. Note that we indicated NA**, whenever social play could not be recorded i.e., for individuals in separation.

#### Observers’ play behaviour

Across conditions and sessions, observers interacted with objects other than the playground setup in 28 instances and for an average of 5.5 s in the experimental phase, when observing one or several individual(s) having access to the playground setup (observers in experimental phase: SD = 22.96, min = 0, max = 252.2; proportion per minute: 0.275), compared to 20 instances for an average of 5.0 s in the baseline phase when no play initiative was provided (observers in baseline phase: SD = 22.78, min = 0, max = 228; proportion per minute: 0.5 s). Moreover, observers showed locomotion play in 83 instances and for an average of 2.9 s in the experimental phase (observers in experimental phase: SD = 6.72, min = 0, max = 61.8; proportion per minute: 0.145 s), compared to 36 instances for an average of 0.9 s in the baseline phase (observers in baseline phase: SD = 2.7, min = 0, max = 19; proportion per minute: 0.09 s). They also showed social play in 20 instances for an average of 2.5 s in the experimental phase (observers in experimental phase, condition 1 only: SD = 8.3, min = 0, max = 56.8; proportion per minute: 0.125 s), compared to five instances for an average of 0.08 s in the baseline phase (observers in baseline phase, condition 1 only: SD = 0.63, min = 0, max = 8; proportion per minute: 0.008 s).

#### Demonstrators’ play behavior

In all trials, the playground setup was manipulated by at least one demonstrator during the experimental phase. Across conditions and sessions, demonstrators interacted with the setup on average for 137.1 s (demonstrators in experimental phase: SD = 169, min = 0, max = 834.6; proportion per minute: 6.9 s). In more detail, they did it for 203.1 s when having access to the playground setup alone (condition 1: SD = 178, min = 9, max = 499; proportion per minute: 10.155 s), as compared to 130.2 s when having access the playground setup within a group setting (condition 2: SD = 167, min = 0, max = 834.6; proportion per minute: 6.51 s; *p* = 0.1055, ns). At three-month post-hatching, the average playground setup manipulation time was 106.8 s (SD = 152.8, min = 0, max = 647; proportion per minute: 5.34 s) as compared to 161.7 s (SD = 177.9 min = 0, max = 834.6; proportion per minute: 8.09 s; *p* = 0.02) at six-month post-hatching.

Note that while having access to the playground setup, demonstrators also engaged in 23 instances of object play with other objects than the playground setup for an average of 2.9 s (demonstrators in experimental phase: SD = 11.4, min = 0, max = 87; proportion per minute: 0.15 s), 83 instances of locomotion play for an average of 2.3 s (demonstrators in experimental phase: SD = 5.7, min = 0, max = 58; across conditions and test ages; proportion per minute: 0.12 s) and they showed 15 instances of social play for an average of 0.6 s (demonstrators in experimental phase, condition 2 only: SD = 2.1, min = 0, max = 12.4; proportion per minute: 0.03 s).

### Effects of others’ object play: models on contagion

To estimate the effect of contagion, we fitted three models, primarily testing for the effect of phase (levels ‘baseline phase’, when no individual had access to the playground setup and ‘experimental phase’ when the demonstrator(s) had access to the playground setup) on the proportion of behaviors of all three play categories expressed by the observer(s), i.e., object play (model 1), locomotion play (model 2) and social play (model 3).

**Fig. 2:**
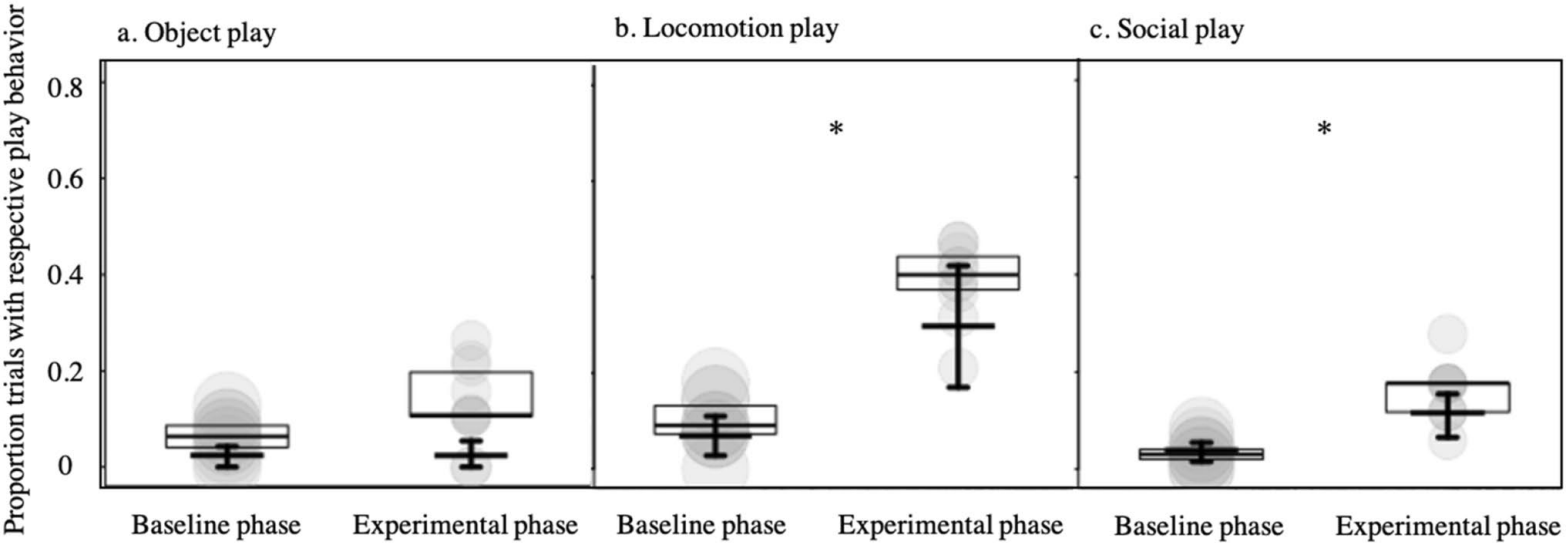
Proportion of trials in which object play (**a**), locomotion play (**b**) and social play (**c**) occurred in the baseline phases (when no individual had access to the playground setup) and the experimental phases (when the demonstrator(s) had access to the playground setup). Indicated are means per observer individual (dots) whereby the area of the dots is proportionate to the number trials per individual and condition (range 17–55). Long thick horizontal lines with boxes indicate medians and quartiles, and short thick horizontal lines with error bars depict the fitted model and its confidence intervals (conditional on all other fixed effects in the model being on their average and an observation duration of 15 min). Note that in **c** the depicted model lacks a random effect of trial ID which, when included, lead to unrealistically low fitted values.

For the object play model (model 1), the full-null model comparison did not reveal significance ($\chi$^*2*^ = 0.519, *df* = 2, *P* = 0.771), and, correspondingly neither the interaction between ‘phase’ and ‘condition’ nor ‘phase’ as a main effect (after removing the interaction) revealed significance (Fig. 2a; Supplementary Tables S1, S2). The full-null model comparisons revealed a clearly significant result in case of the locomotion play model *(*model 2*;* $\chi$^*2*^ = 15.639, *df* = 2, *p* < 0.001). However, the interaction between ‘condition’ and ‘phase’ did not reveal significance ($\chi$^*2*^ = 1.213, *df* = 1, *P* = 0.271; Supplementary Table S3) and hence we removed it from the model. The reduced model revealed a clearly significant result for ‘phase’ (Supplementary Table S4), whereby the observer individual(s) showed more locomotion play during the experimental phase, when the demonstrator individual(s) had access to the playground setup in comparison to the baseline phase, when no play initiative was provided (Fig. 2b). The social play model (model 3) also revealed a significant effect of ‘phase’ ($\chi$^*2*^ = 5.198, *df* = 1, *P* = 0.023) as social play was more likely to occur during the experimental phase in comparison to the baseline phase (Fig. 2c; Supplementary Table S5). The age of the birds (three-month or six-month post-hatching) was not a significant factor in any of our models.

### First play behavior of observers

Following Osvath and Sima (2014), we checked the very first play behavior of each trial that an observer individual engaged in after the demonstrator(s) had started manipulating the playground setup. In 23 cases (85.2%), we recorded locomotion play as an observer’s initial play behavior. In contrast, we recorded only three cases of object play (11.1%) and only one case of social play (3.7%) as a first response. All three cases of object play and the single case of social play as first responses occurred during the second test session at the age of six-month post-hatching.

### Effect of playground setup: ghost control condition

The proportions observers spent with object-, locomotion- and social play per minute during the experimental phase of the ghost control condition (playground setup presented without a demonstrator during the experimental phase) were relatively low both in comparison to the respective proportions of play in the baseline phases of the ghost control condition (Supplementary table S6), and also in comparison to the respective proportions of play in the experimental phases of condition 1 and 2 (with the only exception being the proportion of object play in session 2; Table 1 and Supplementary table S6).

## Discussion

In the present study, we aimed at assessing developmental trajectories of behavioral and emotional contagion in raven play by studying ten juvenile individuals at the age of three- and six-month post-hatching. We reliably elicited object play in one or more birds using a playground setup and recorded the play behavior of the remaining conspecific(s), who acted as observers. Compared to the baseline phase, observers showed an increase of locomotion play and social play during the experimental phase, when the demonstrator(s) had access to the playground setup; however, observers did not increase their object play. These patterns were found at both testing times, i.e., at three- and six-month post-hatching, and closely resemble the patterns found in a previous study by Osvath and Sima (2014) on older ravens.To our knowledge, hardly any studies have experimentally addressed the developmental trajectories of behavioral and emotional contagion in animals (but see studies on contagion yawning for developmental effects:Anderson et al. 2004; Palagi et al. 2009). That is why we tested our ravens at two test times, at the beginning and end of their first summer, at three-month and six-month post-hatching. As Osvath and colleagues found indications of behavioral mimicry in three raven nestlings (2014), we expected our subjects to be capable of behavioral contagion already during the first test session at three-month post-hatching. Based on previous ontogenetic studies that indicate crucial developmental steps towards more complex socio-cognitive behaviors during the first summer of a raven’s life (e.g., capacities that include perspective taking, (Bugnyar et al. 2007; Schloegl et al. 2007), we expected emotional contagion to be present only after the ravens’ first summer, in the second test session at six-month post-hatching. Following the widely recognized definition of behavioral mimicry as a precursor of emotional contagion (Preston and de Waal 2002; Chartrand and van Baaren 2009; Feneran et al. 2013), we also expected behavioral contagion to occur during the second test session. Hence, behavioral contagion should be found in both test periods, before and after summer while emotional contagion was expected to occur only after summer. Contrary to our expectations, we did not see indications for behavioral contagion but evidence for emotional contagion at both test sessions, i.e., at three- and six-month post-hatching. In either session, observer ravens hardly copied motor patterns from demonstrator individuals but got into a general play mood. In line with previous research (Osvath et al. 2014), we interpret the observers’ behavior as evidence for emotional contagion. When analyzing the very first play behavior of each trial that an observer engaged in, we found additional descriptive support for the expression of emotional contagion: in 88.9% of the cases, the first play behavior in observer ravens was locomotion play or social play, while object play was the first behavior in only 11.1% of the cases. Thus, ravens as young as three months were already capable to pick up and align to emotional states of their conspecifics in a play context. Possibly, this first stage of empathic ability serves as an important building block in early social life, e.g., for acquiring skills or social competence.Interestingly, we did not find any effect of behavioral contagion as the ravens did not engage in more object play during the experimental phase in comparison to the baseline phase. Note that our measure of ‘object play’ contained manipulation of items in the ravens’ natural environment, including aviary equipment like sticks; we can thus rule out that the observed rates of object play were limited by the availability of material. It is possible, however, that the ravens copied the play behavior of the demonstrator(s) not so much in respect to object manipulation but in respect to body postures and behavioral sequences, which our measurements did not pick up. Although we regard this possibility as unlikely, future studies should take the topography of behaviors and their sequences into account (compareVoelkl and Huber, 2000, 2007; Huber et al. 2009). According to the definition of emotional contagion (Hatfield 1994), behavioral synchronization is a pre-step towards an emotional alignment. Finding support for emotional but not behavioral contagion in our data, thus, questions the role of behavioral matching as a precursor for emotional contagion. Within the human literature, many studies were unable to establish a direct connection between the synchronization of facial expressions and a resulting emotional experience that was comparable to the demonstrators’ (e.g., Hess and Blairy 2001). Furthermore, recent studies in non-human animals reported cases of emotional contagion without any simultaneous behavioral contagion (Adriaense et al. 2019; Isern-Mas and Gomila 2019). For instance, sub/adult ravens show a pessimistic judgement bias in response to observing others in distress or frustration, without showing any behavioral expressions indicative for distress or frustration themselves (Adriaense et al. 2019). Hence, our current results support the assumption that emotional contagion does not rest on behavioral contagion but reflects an independent system (Singer and Lamm 2009; Yamamoto 2017; Edgar and Nicol 2018; Adriaense et al. 2020) that develops early in life. In comparison to previous work (Osvath et al. 2014), we applied a more systematic experimental procedure and improved the diversity of demonstrators by separating one individual per trial. By looking at the observers’s behavior when confronted with either a single, or multiple demonstrators, we could also account for the strength of potential contagion effects. However, our models did not show differences in the contagion effects of individuals confronted with a single or multiple demonstrator(s). The occurrence of emotional contagion was therefore not affected by the number of demonstrators; indicating, that play behavior of a single conspecific sufficiently triggered play behavior and emotional contagion in observer birds. Moreover, we found that the presentation of the playground setup alone did not lead to an increase of locomotion or social play in a ghost control condition, indicating that it was the demonstrator(s)’s interaction with the playground setup, and not the playground setup itself, that elicited play in the observers. An interesting fact was the large variation in whether ravens did or did not engage in play (compare the relatively frequent occurrences of zero play, see statistical data analysis), which could be due to several internal and external factors. For instance, young ravens have relatively short activity bouts after fledging (Heinrich and Smolker 1998), which might explain different levels of motivation to engage in object manipulation and play as well as their social attention. Their motivation to play might also be affected by environmental conditions like hot weather and their immediate social surrounding. For future research, it would be interesting to consider the role of hierarchy or individual social bonds when looking at contagion effects. In line with previous literature (Kavaliers et al. 2001, 2005; Jeon et al. 2011; Martin et al. 2015), we gained the impression, that more attention was paid to high-ranking and closely bonded demonstrators and therefore, play mood could have been easier transferred between individuals (personal observation). Nevertheless, assessing the effects of kinship, social bonds and dominance hierarchy was beyond the scope of this study due to the limited sample size. Taken together, when we experimentally elicited object play in one or more juvenile ravens using a playground setup, we found evidence for emotional contagion at both testing ages at three- and six-month post-hatching, as observer ravens showed an increase of locomotion play and social play as a response to experiencing conspecific(s) having access to a playground setup nearby. However, we did not see indicators for behavioral contagion, as no increase of object play occurred at either test age. Our findings thus speak against behavioral contagion as a precondition for emotional contagion. That emotional contagion occurs in raven play from a very early age on may hint towards the importance of empathic-like building blocks in social life, e.g., for acquiring skills or social competence.

## Supplementary Information

Below is the link to the electronic supplementary material.

Supplementary file1 (DOCX 29 KB)
