# Supplementary material for: Early evidence for emotional play contagion in juvenile ravens
Source: Anim Cogn. 2021 Jan 9;24(4):717–29. doi: 10.1007/s10071-020-01466-0 (PMC8238721; doi:10.1007/s10071-020-01466-0)
Supplement: Supplementary file 1 — Supplementary file1 (DOCX 29 KB) [file 10071_2020_1466_MOESM1_ESM.docx]

**Early evidence for emotional play contagion in juvenile ravens**

Katharina Wenig*^1,2,3^, Palmyre H. Boucherie^1,4^, Thomas Bugnyar^1,2,5^

^1^ Department of Behavioral and Cognitive Biology, University of Vienna, Austria

^2^ Haidlhof Research Station, University of Vienna and University of Veterinary Medicine Vienna

^3^ e-mail: katharina.wenig@univie.ac.at

^4^ e-mail: palmyre.boucherie@univie.ac.at

^5^ e-mail: thomas.bugnyar@univie.ac.at

*Correspondence should be addressed to

Katharina Wenig

Department of Behavioral and Cognitive Biology

Althanstrasse 14

1090 Vienna, Austria

e-mail: katharina.wenig@univie.ac.at

Supplementary Table S1. Results of the full model investigating object play (binary response variable). Estimates are provided together with standard errors, confidence intervals, confidence limits, significance tests, and the range of estimates when excluding levels of the random effects one at a time.

| term | Estimate | SE | lower Cl | upper Cl | χ^2^ | df | P | min | max |
| --- | --- | --- | --- | --- | --- | --- | --- | --- | --- |
| intercept | -6.635 | 0.569 | -12.911 | -5.945 |  |  | ^(1)^ | -7.133 | -6.371 |
| phase^(2)^ | 0.276 | 0.793 | -2.133 | 2.469 |  |  | ^(1)^ | -0.082 | 0.642 |
| condition^(3)^ | 2.042 | 0.538 | 0.426 | 3.936 |  |  | ^(1)^ | 1.713 | 2.344 |
| age^(4)^ | -0.120 | 0.618 | -1.971 | 1.784 |  |  | ^(1)^ | -0.461 | 0.375 |
| trial nr.^(5)^ | 0.476 | 0.326 | -0.168 | 1.869 |  |  | ^(1)^ | 0.364 | 0.615 |
| phase:condition | -0.914 | 1.307 | -11.099 | 2.363 | 0.519 | 1 | 0.471 | -2.016 | -0.007 |
| age:trial nr. | -0.249 | 0.660 | -2.457 | 1.613 | 0.141 | 1 | 0.707 | -0.517 | 0.075 |

^(1)^ not indicated because of having a very limited interpretation

^2)^ dummy coded with ‘baseline phase’ being the reference level

^(3)^ dummy coded with ‘condition 1’ (multiple observers in a group setting, being confronted with a single demonstrator) being the reference level

^(4)^ centered to a mean of zero

^(5)^ z-transformed to a mean of zero and a standard deviation of one; mean and standard deviation of the original variable were 6.618 and 3.532, respectively

Supplementary Table S2. Results of the reduced model (lacking the interaction between ‘phase’ and ‘condition’) investigating object play (binary response variable). Estimates are provided together with standard errors, confidence intervals, confidence limits, and significance tests.

| term | Estimate | SE | lower Cl | upper Cl | χ^2^ | df | P |
| --- | --- | --- | --- | --- | --- | --- | --- |
| intercept | -6.592 | 0.564 | -17.256 | -5.877 |  |  | ^(1)^ |
| phase^(2)^ | -0.012 | 0.716 | -2.726 | 2.460 | <0.001 | 1 | 0.988 |
| condition^(3)^ | 1.890 | 0.506 | 0.084 | 5.840 | 7.290 | 1 | 0.007 |
| age^(4)^ | -0.077 | 0.622 | -1.851 | 1.936 |  |  | ^(1)^ |
| trial nr.^(5)^ | 0.490 | 0.326 | -0.291 | 1.819 |  |  | ^(1)^ |
| age:trial nr. | -0.241 | 0.666 | -2.573 | 1.505 | 0.129 | 1 | 0.719 |

^(1)^ not indicated because of having a very limited interpretation

^2)^ dummy coded with ‘baseline phase’ being the reference level

^(3)^ dummy coded with ‘condition 1’ (multiple observers in a group setting, being confronted with a single demonstrator) being the reference level

^(4)^ centered to a mean of zero

^(5)^ z-transformed to a mean of zero and a standard deviation of one; mean and standard deviation of the original variable were 6.618 and 3.532, respectively

Supplementary Table S3. Results of the full model investigating locomotion play (binary response variable). Estimates are provided together with standard errors, confidence intervals, confidence limits, significance tests and the range of estimates when excluding levels of the random effects one at a time.

| term | Estimate | SE | lower Cl | upper Cl | χ^2^ | df | P | min | max |
| --- | --- | --- | --- | --- | --- | --- | --- | --- | --- |
| intercept | -5.361 | 0.338 | -6.375 | -4.839 |  |  | ^(1)^ | -5.546 | -5.189 |
| phase^(2)^ | 1.808 | 0.416 | 1.091 | 2.889 |  |  | ^(1)^ | 1.646 | 1.969 |
| condition^(3)^ | 1.356 | 0.658 | -0.818 | 2.681 |  |  | ^(1)^ | 0.978 | 1.695 |
| age^(4)^ | -0.428 | 0.463 | -1.477 | 0.567 |  |  | ^(1)^ | -0.684 | -0.085 |
| trial nr.^(5)^ | 0.422 | 0.209 | 0.017 | 0.941 |  |  | ^(1)^ | 0.332 | 0.510 |
| phase:condition | -1.167 | 1.019 | -4.913 | 1.461 | 1.213 | 1 | 0.271 | -1.841 | -0.645 |
| age:trial nr. | -1.730 | 0.434 | -2.902 | -0.992 | 13.949 | 1 | <0.001 | -1.927 | -1.545 |

^(1)^ not indicated because of having a very limited interpretation

^(2)^ dummy coded with ‘baseline phase’ being the reference level

^(3)^ dummy coded with ‘condition 1’ (multiple observers in a group setting, being confronted with a single demonstrator) being the reference level

^(4)^ centered to a mean of zero

^(5)^ z-transformed to a mean of zero and a standard deviation of one; mean and standard deviation of the original variable were 6.618 and 3.532, respectively

Supplementary Table S4. Results of the reduced model (lacking the interaction between ‘phase’ and ‘condition’) investigating locomotion play (binary response variable). Estimates are provided together with standard errors, confidence intervals, confidence limits, and significance tests.

| term | Estimate | SE | lower Cl | upper Cl | χ^2^ | df | P |
| --- | --- | --- | --- | --- | --- | --- | --- |
| intercept | -5.405 | 0.356 | -6.448 | -4.924 |  |  | ^(1)^ |
| phase^(2)^ | 1.742 | 0.428 | 1.068 | 2.821 | 14.426 | 1 | <0.001 |
| condition^(3)^ | 0.984 | 0.743 | -0.928 | 2.380 | 1.595 | 1 | 0.207 |
| age^(4)^ | -0.355 | 0.479 | -1.410 | 0.620 |  |  | ^(1)^ |
| trial nr.^(5)^ | 0.393 | 0.217 | 0.048 | 0.892 |  |  | ^(1)^ |
| age:trial nr. | -1.792 | 0.455 | -3.074 | -1.086 | 13.167 | 1 | <0.001 |

^(1)^ not indicated because of having a very limited interpretation

^(2)^ dummy coded with ‘baseline phase’ being the reference level

^(3)^ dummy coded with ‘condition 1’ (multiple observers in a group setting, being confronted with a single demonstrator) being the reference level

^(4)^ centered to a mean of zero

^(5)^ z-transformed to a mean of zero and a standard deviation of one; mean and standard deviation of the original variable were 6.618 and 3.532, respectively

Supplementary Table S5. Results of the model investigating social play (binary response variable). Estimates are provided together with standard errors, confidence intervals, confidence limits, significance tests, and the range of estimates when excluding levels of the random effects one at a time.

| term | Estimate | SE | lower Cl | upper Cl | χ^2^ | df | P |
| --- | --- | --- | --- | --- | --- | --- | --- |
| intercept | -7.166 | 0.678 | -9.853 | -6.305 |  |  | ^(1)^ |
| phase^(2)^ | 1.573 | 0.694 | 0.128 | 3.149 | 5.198 | 1 | 0.023 |
| age ^(3)^ | -0.097 | 0.717 | -1.674 | 1.755 |  |  | ^(1)^ |
| trial nr.^(4)^ | 0.953 | 0.405 | 0.246 | 1.899 |  |  | ^(1)^ |
| age:trial nr. | -0.906 | 0.786 | -2.639 | 0.595 | 1.424 | 1 | 0.233 |

^(1)^ not indicated because of having a very limited interpretation

^(2)^ dummy coded with ‘baseline phase’ being the reference level

^(3)^ centered to a mean of zero

^(4)^ z-transformed to a mean of zero and a standard deviation of one; mean and standard deviation of the original variable were 6.610 and 3.521, respectively

Supplementary Table S6. Average play behaviors in seconds (and corresponding standard deviations, minimum and maximum values and proportions per minute of the respective phase, i.e., 10 minutes in the baseline phase, 20 minutes in the experimental phase; format: Mean (SD/Min/Max/**Proportion**)) for all observers individuals within sessions (session 1: 3-months post-hatching, session 2: 6-months post-hatching) and test phase (baseline phase, experimental phase) in the ghost control condition: i.e., playground setup was placed into the separation compartment during the experimental phase, but no individual was separated as a demonstrator; all individuals were therefore in group setting as observers; baseline phases in the ghost control condition were identical to the baseline phases in the other tested conditions).

|  | | Ghost Control Condition |  |
| --- | --- | --- | --- |
| Object  play | Baseline phase | 5.4 (16.3/0/70/**0.5**) | Session 1 |
|  |  | 7.9 (58.9/0/437/**0.8**) | Session 2 |
|  | Experimental phase | 1.7 (9.7/0/68/**0.08**) | Session 1 |
|  |  | 9.4 (42.2/0/287/**0.5**) | Session 2 |
| Locomotion play | Baseline phase | 1.4 (4/0/25/**0.1**) | Session 1 |
|  |  | 1.15 (3.7/0/23/**0.11**) | Session 2 |
|  | Experimental phase | 1.5 (3/0/13/**0.08**) | Session 1 |
|  |  | 0.95 (5.1/0/38/**0.05**) | Session 2 |
| Social  play | Baseline phase | 0 (0/0/0/**0**) | Session 1 |
|  |  | 0 (0/0/0/**0**) | Session 2 |
|  | Experimental phase | 0 (0/0/0/**0**) | Session 1 |
|  |  | 0 (0/0/0/**0**) | Session 2 |
